# Seronegative myasthenic crisis: a multicenter analysis

**DOI:** 10.1007/s00415-022-11023-z

**Published:** 2022-04-07

**Authors:** Philipp Mergenthaler, Henning R. Stetefeld, Christian Dohmen, Siegfried Kohler, Silvia Schönenberger, Julian Bösel, Stefan T. Gerner, Hagen B. Huttner, Hauke Schneider, Heinz Reichmann, Hannah Fuhrer, Benjamin Berger, Jan Zinke, Anke Alberty, Ingo Kleiter, Christiane Schneider-Gold, Christian Roth, Juliane Dunkel, Andreas Steinbrecher, Andrea Thieme, De-Hyung Lee, Ralf A. Linker, Klemens Angstwurm, Andreas Meisel, Bernhard Neumann, for The German Myasthenic Crisis Study Group

**Affiliations:** 1 Charité - Universitätsmedizin Berlin, Department of Neurology with Experimental Neurology, Neuroscience Clinical Research Center, Berlin, Germany; 2 Charité - Universitätsmedizin Berlin, Center for Stroke Research Berlin, Berlin, Germany; 3 Berlin Institute of Health at Charité-Universitätsmedizin Berlin, BIH Academy, 10117 Berlin, Germany; 4 Faculty of Medicine, Department of Neurology, University of Cologne, University Hospital Cologne, Cologne, Germany; 5 Department of Neurology, LVR-Klinik Bonn, Bonn, Germany; 6 Department of Neurology, Heidelberg University Hospital, Heidelberg, Germany; 7 Department of Neurology, Klinikum Kassel, Kassel, Germany; 8 Department of Neurology, University Hospital Erlangen, Erlangen, Germany; 9 Department of Neurology, Universitätsklinikum Gießen Und Marburg, Giesen, Germany; 10 Department of Neurology, University Hospital, Technische Universität Dresden, Dresden, Germany; 11 Department of Neurology, University Hospital Augsburg, Augsburg, Germany; 12 Clinic of Neurology and Neurophysiology, Medical Center-University of Freiburg, Faculty of Medicine, University of Freiburg, Freiburg, Germany; 13 Hans Berger Department of Neurology, Jena University Hospital, Jena, Germany; 14 Department of Neurology, Kliniken Maria Hilf GmbH Moenchengladbach, Mönchengladbach, Germany; 15 Department of Neurology, St. Josef-Hospital, Ruhr-University Bochum, Bochum, Germany; 16 Marianne-Strauß-Klinik, Behandlungszentrum Kempfenhausen Für Multiple Sklerose Kranke gGmbH, Berg, Germany; 17 Department of Neurology, DRK-Kliniken Nordhessen, Kassel, Germany; 18 Department of Neurology, Philipps University of Marburg, Marburg, Germany; 19 Department of Neurology, Helios Klinikum Erfurt, Erfurt, Germany; 20 Department of Neurology, University of Regensburg, Bezirksklinikum, Regensburg, Germany; 21 Berlin Institute of Health at Charite-Universitatsmedizin Berlin, Berlin, Germany; 22 Present Address: Department of Neurology, Donau-Isar-Klinikum Deggendorf, Deggendorf, Germany

**Keywords:** Myasthenia gravis, Myasthenic crisis, Antibody status, Seronegative, Outcome

## Abstract

Myasthenic crisis (MC) is a life-threatening condition for patients with myasthenia gravis (MG). Seronegative patients represent around 10–15% of MG, but data on outcome of seronegative MCs are lacking. We performed a subgroup analysis of patients who presented with MC with either acetylcholine-receptor-antibody-positive MG (AChR-MG) or seronegative MG between 2006 and 2015 in a retrospective German multicenter study. We identified 15 seronegative MG patients with 17 MCs and 142 AChR-MG with 159 MCs. Seronegative MCs were younger (54.3 ± 14.5 vs 66.5 ± 16.3 years; *p* = 0.0037), had a higher rate of thymus hyperplasia (29.4% vs 3.1%; *p* = 0.0009), and were more likely to be female (58.8% vs 37.7%; *p* = 0.12) compared to AChR-MCs. Time between diagnosis of MG and MC was significantly longer in seronegative patients (8.2 ± 7.6 vs 3.1 ± 4.4 years; *p* < 0.0001). We found no differences in duration of mechanical ventilation (16.2 ± 15.8 vs 16.5 ± 15.9 days; *p* = 0.94) and length of stay at intensive care unit (17.6 ± 15.2 vs 17.8 ± 15.4 days; *p* = 0.96), or in-hospital mortality (11.8% vs. 10.1%; *p* = 0.69). We conclude that MC in seronegative MG affects younger patients after a longer period of disease, but that crisis treatment efficacy and outcome do not differ compared to AChR-MCs.

## Introduction

Myasthenia gravis (MG) is an autoimmune disease with antibodies (Abs) targeting the postsynaptic neuromuscular junction. Ultimately, muscle fatigability and weakness are caused by disrupted neuromuscular signaling. Nearly 90% of all MG patients have positive test results for AChR, Muscle-specific kinase (MuSK), or low-density lipoprotein receptor-related protein (LRP4) autoantibodies, with the majority tested positive for AChR-Abs [1]. However, in around 10–15% of MG patients no specific autoantibodies can be found. This group of seronegative patients is also thought to include patients with very low antibody titers, low-affinity antibodies and yet to be defined autoantigens [1].

Myasthenic crisis (MC) is the most severe form of MG and is potentially life threatening. MC is mostly provoked by infections, but also fever, aspiration, inadequate treatment, various medications, or following surgery [2]. In the first two years after diagnosis, around 15–20% of MG patients suffer from a MC [2, 3]. Characteristic symptoms are extensive weakness, dysphagia, and dyspnea which can result in respiratory insufficiency. The clinical management of MC is well defined and has led to a significant decline in mortality from around 40% in the early 1960s to 5% to 12% in recent studies [2–8].

However, to date little is known about the management of MC in seronegative MG. Here, we therefore investigated seronegative patients with MC and compared their crises to AChR-MCs regarding clinical features, therapeutic management, and outcome.

## Methods

### Study design and patient selection

We performed a subgroup analysis of seronegative MC needing mechanical ventilation (MV) compared to AChR-MC treated at eight German Departments of Neurology with specialized Neuro-Intensive Care Units (NICU) or neurologically associated interdisciplinary ICU [2]. For identification, records of all patients discharged with the diagnosis of MG according to the International Classification of Diseases (ICD10: G70.0–70.3) who were treated and ventilated on an ICU between 2006 and 2015 were reviewed. MC was defined as an exacerbation of myasthenic symptoms with bulbar and/or general weakness requiring MV. Seronegative MG was defined as absence of AChR and MuSK autoantibodies. Per protocol, antibody status was confirmed by routine laboratory testing using certified assays. Most AChR-Abs and MuSK-Abs were tested by radio-receptor assay, but the method is not known in all cases due to the retrospective character. Diagnosis of MG had to be established clinically according to national guidelines and confirmed by specific tests (antibody testing or repetitive stimulation or improvement after cholinergic medication) [9]. New episodes of MC were counted separately if patients were discharged in their prehospital status and if new triggers for the next crisis could be determined. For this analysis, we only included AChR-MCs treated at the same centers as the seronegative MCs to reduce treatment and data acquisition bias.

### Data acquisition

Data on baseline demographics, clinical information, medication and comorbidities were obtained through review of medical records and institutional databases. Characteristics reviewed included antibody-status, evidence of thymoma and Myasthenia Gravis Foundation of America (MGFA) Score prior to MC. Assessed treatment regimens were intravenous immunoglobulins (IVIG), plasma exchanging therapy (PE), immunoadsorption (IA), use of intravenous pyridostigmine, and continuous potassium infusion. Analyzed data regarding the MC included time at intensive care unit (ICU-LOS), days in hospital, duration of MV, in-hospital mortality and referral/discharge. In addition, we performed a survey about LRP4- and Agrin-antibody-positive MGs in our study group in June 2021.

### Statistics

GraphPad Prism 5® (GraphPad Software, La Jolla, USA) was used for statistical analysis. Data were presented as mean with standard deviation or range (as indicated) or total number with percentage. Group comparison was tested with either Student’s *t* test or Fisher’s exact test (with odds ratios (OR)). The significance level was set to α = 0.05 both sided.

## Results

### Characteristics of study group

**Fig. 1:**
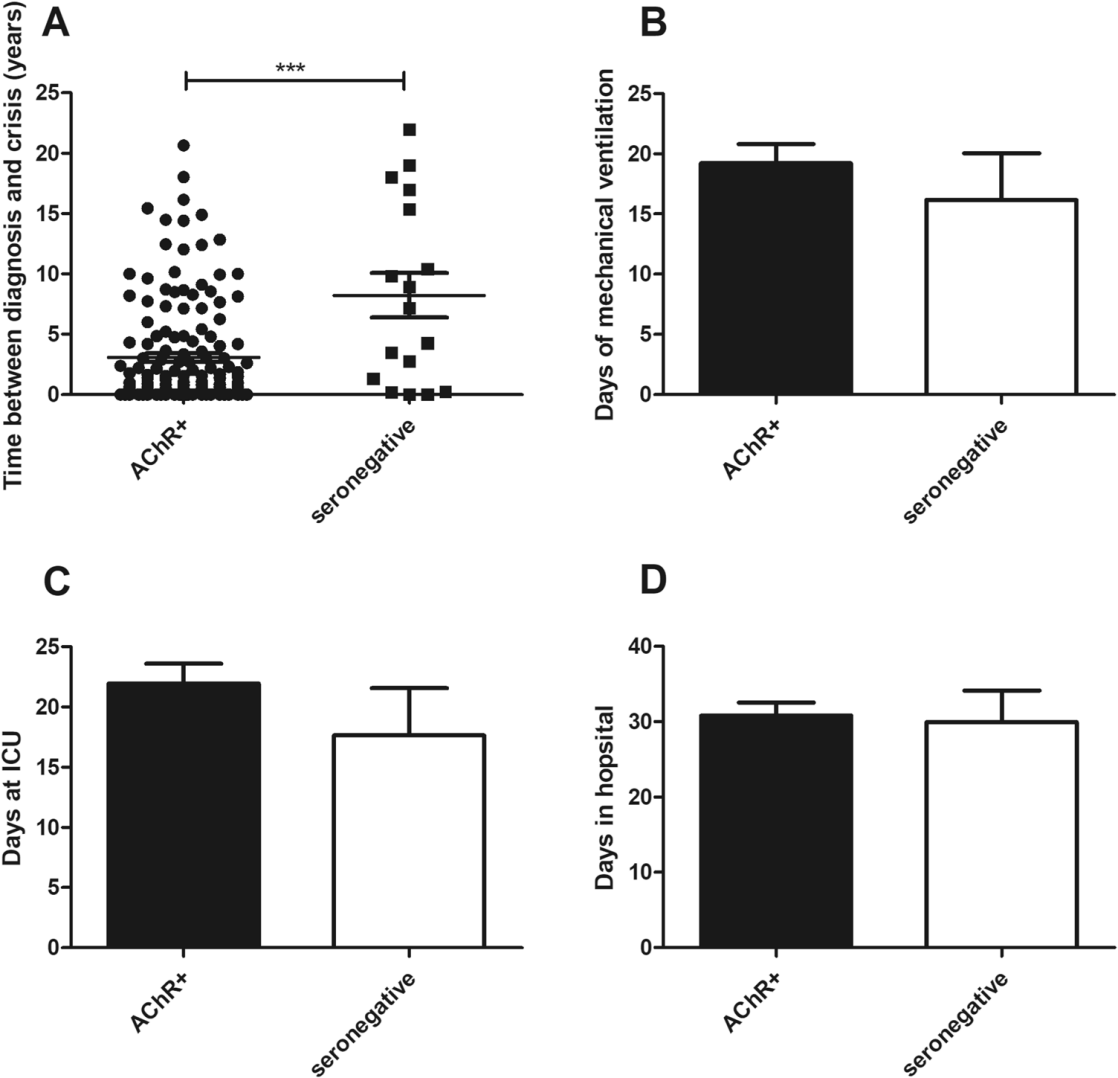
**A** Time between first diagnosis of MG to first MC in years. Every dot or square symbolizes one patient. Long line shows mean, short lines show SD (*t* test). ***p < 0.001 **B** Days of mechanical ventilation, **C** Days at ICU, **D** Days in hospital in 159 MCs with AChR-Abs and 17 MCs with MuSK-Abs. **B–D** Bars show mean ± SD (*t* test). No significant results were found

The cohort consisted of 15 patients with 17 seronegative MCs and 142 AChR antibody-positive patients with 159 MCs requiring MV. Patients from both groups were treated at the same centers (Table 1). Seronegative patients were responsible for 6.8% of the crises in our whole cohort (*n* = 250 crises). Patients with seronegative MC were significantly younger (54.3 ± 14.5 vs 66.5 ± 16.3; *p* = 0.0037) and more likely to be female (58.8 vs 37.7%; *p* = 0.12) than AChR-MCs. AChR-MCs were significantly more often late-onset MGs (85.5% vs 41.2%; *p* = 0.0001; OR = 0.12), whereas seronegative MCs belonged mainly to the early-onset group (Table 1) and had significantly more frequently a thymus hyperplasia (29.4% vs 3.1%; *p* = 0.0009; OR 12.83). Thymus hyperplasias were resected in all patients prior to crisis, except in one patient in the AChR-group. Importantly, the time between diagnosis of MG and onset of MC was significantly longer in seronegative patients (8.2 ± 7.6 vs 3.1 ± 4.4 years; *p* < 0.0001) (Fig. 1A). Due to the higher age, patients with AChR-MCs had more comorbidities, yet without reaching statistical significance (Table 1). We also did not find statistically significant differences in the status before crisis, MGFA classification before crisis, dosage of pyridostigmine treatment before crisis (252.4 ± 243.3 vs 251.1 ± 206.6 mg/d; *p* = 0.99) or number of myasthenic worsening/crises before present MC (Table 1). The number of days between first symptoms of MC and hospitalization were similar (9.9 ± 13.1 vs 9.6 ± 14.9; *p* = 0.95).

**Table 1:** Comparison of episodes of myasthenic crisis with AChR-Abs and seronegative patients

Myasthenic crises	AChR-positive (*n* = 159)	Seronegative (*n* = 17)	*P* value	Odds ratio
**Age**	**66.5 ± 16.3 (14–89)**	**54.3 ± 14.5 (25–81)**	**0.0037**	
Male/female	99 (62.3%)/60 (37.7%)	7 (41.2%)/10 (58.8%)	0.12	0.42
Pulmonary disease	38 (23.9%)	2 (11.8%)	0.37	0.42
Heart disease	61 (38.4%)	5 (29.4%)	0.60	0.67
Diabetes mellitus	48 (30.2%)	2 (11.8%)	0.16	0.31
Tumour (other than thymoma)	24 (15.1%)	0 (0%)	0.13	0.16
Dialysis	1 (0.6%)	0 (0%)	1.00	3.02
Smoker	16 (10.1%)	2 (11.8%)	0.69	1.19
Alcohol addicted	5 (3.1%)	2 (11.8%)	0.14	4.11
≥ 3 diseases (kidney, heart, lung, diabetes, tumour)	21 (13.2%)	0 (0%)	0.23	0.18
Myasthenia gravis				
**Early onset**	**22 (13.8%; 1 unknown)**	**10 (58.8%)**	** < 0.0001**	**8.90**
**Late onset**	**136 (85.5%)**	**7 (41.2%)**	**0.0001**	**0.12**
Paraneoplastic MG (Thymoma)	53 (33.3%)	3 (17.6%)	0.27	0.43
** Thymus hyperplasia**	**5 (3.1%)**	**5 (29.4%)**	**0.0009**	**12.83**
Number of myasthenic worsenings/crises before present myasthenic crisis	0.7 ± 1.3	0.9 ± 1.3	0.49	
** Time between first diagnosis and crisis (years)**	**3.1 ± 4.4 (0–18.2)**	**8.2 ± 7.6 (0–22)**	** < 0.0001**	
MGFA-classification before crisis				
First manifestation of MG	38 (23.9%)	3 (17.6%)	0.77	0.68
Class I	8 (5.0%)	1 (5.9%)	1.00	1.18
Class II	44 (27.7%)	4 (23.5%)	1.00	0.80
Class III	40 (25.2%)	4 (23.5%)	1.00	0.92
Class IV	16 (10.1%)	2 (11.8%)	0.69	1.19
Unknown	13 (8.2%)	2 (11.8%)		
Status before crisis				
Independent at home	73 (45.9%)	7 (41.2%)	0.80	0.82
At home dependent on help	23 (14.5%)	3 (17.6%)	0.72	1.27
In a care facility or hospital	49 (30.8%)	7 (41.2%)	0.42	1.57
Unknown	14 (8.8%)	0 (0%)		
Cause of crisis				
Infection	80 (50.3%)	8 (47.1%)	n.s	
First episode	36 (22.6%)	3 (17.6%)		
Poor treatment compliance	14 (8.8%)	0 (0%)		
Intake of contraindicated medication	2 (1.2%)	0 (0%)		
Idiopathic/unknown	32 (20.1%)	6 (35.3%)		
Therapy				
**IVIG**	**93 (58.5%)**	**4 (23.5%)**	**0.009**	**0.22**
Plasma exchange/immunoadsorption in total	75 (47.2%)	10 (58.8%)	0.45	1.60
PE or IA as first line therapy	60 (37.7%)	10 (58.8%)	0.12	2.34
IVIG + plasma exchange or immunoadsorption	30 (18.9%)	1 (5.9%)	0.31	0.27
Continuous pyridostigmine infusion	61 (38.4%)	5 (29.4%)	0.60	0.67
Complications				
CPR	19 (11.9%)	2 (11.8%)	1.00	0.98
Pneumonia	86 (54.1%)	9 (52.9%)	1.00	0.95
Sepsis	32 (20.1%)	3 (17.6%)	1.00	0.85
Outcome				
Days of mechanical ventilation at ICU	19.2 ± 19.5 (1–119)	16.2 ± 15.8 (1–55)	0.54	
Days at ICU	22.0 ± 20.5 (1–135)	17.6 ± 15.2 (3–56)	0.42	
Days in hospital	30.8 ± 21.4 (3–144)	29.9 ± 16.5 (3–71)	0.87	
In-hospital mortality	16 (10.1%)	2 (11.8%)	0.69	1.19

Age, “Days of mechanical ventilation at ICU”, “Days at ICU”, “Days in hospital” and “Time between first diagnosis and crisis (years)”are depicted as mean ± Standard Deviation and range, other parameters are total number with percentage in brackets. *MGFA* Myasthenia Gravis Foundation of America, *MG* Myasthenia Gravis, *IVIG* Intravenous Immunoglobulin, *PE* Plasma exchange, *IA* Immunoadsorption, *CPR* Cardio Pulmonal Resuscitation, *n.s.* not significant. *t* test was used for statistical analysis of age-differences and for comparison of “Days of mechanical ventilation at ICU”, “Days at ICU” and “Days in hospital”. For other parameters Fisher`s exact test with odds ratio was used. Significant result (*p* ≤ 0.05) are shown in bold letters

To further characterize the patients with seronegative MCs, we surveyed all participating centers on retesting for LRP4 and Agrin antibodies in these seronegative patients. 6 of 15 seronegative MGs were tested for LRP4-antibodies and 5 of 15 for Agrin-antibodies. However, all tests remained negative. At the centers of our study group 28 patients with LRP4 and 0 patients with Agrin-antibodies are treated, but none developed an MC needing ICU-treatment within the period of observation.

### Treatment and outcome

Seronegative MCs were significantly less frequently treated with IVIGs (23.5% vs 58.5%; *p* = 0.009; OR = 0.22) (Table 1), and although they received PE or IA more frequently than AChR-MCs, no statistically significant difference could be found (58.8% vs 47.2%; *p* = 0.45; OR = 1.60). Likewise, although AChR-MCs were more frequently treated with the combination of PE or IA and IVIG, this was not statistically significant (18.9% vs 5.9%; *p* = 0.31; OR = 0.27). Furthermore, days of MV at ICU (16.2 ± 15.8 vs 19.2 ± 19.5; *p* = 0.54), ICU-LOS (17.6 ± 15.2 vs 22.0 ± 20.5; *p* = 0.42) and hospital-LOS (29.9 ± 16.5 vs 30.8 ± 21.4; *p* = 0.87) were not statistically significantly different (Table 1 and Fig. 1B-D). The in-hospital mortality was similar between both groups (11.8% vs 10.1%; *p* = 0.69; OR = 1.19). Importantly, seronegative patients were less frequently discharged while still needing MV (5.9% vs 20.8%; *p* = 0.20; OR = 0.24) compared to AChR-MG patients, but without a statistically significant difference. Consequently, after matching 17 seronegative to 51 AChR-positive MCs regarding age (54.3 ± 14.5 vs 53.9 ± 16.3; *p* = 0.93) and sex (58.8% vs 53.0% female; *p* = 0.78) we found no differences in days of MV (16.2 ± 15.8 vs 16.5 ± 15.9; *p* = 0.94) and ICU-LOS (17.6 ± 15.2 vs 17.8 ± 15.4; *p* = 0.96). Treatment and outcome details for all 17 seronegative MCs are shown in Table 2. We found no difference between the treatment options IVIG and PE/IA or the additional use of intravenous pyridostigmine regarding the endpoint duration of MV in seronegative MCs.

**Table 2:** Treatment and outcome details of all 17 seronegative MCs

Patient no.	Age	IVIG	PE/IA	Pyridostigmine intravenous	Days of mechanical ventilation	Death
1	80	5 × 30 g	No	No	38	No
2	66	No	5 cycles of PE	10,8 mg/24 h	39	No
3	25	No	5 cycles of PE	9,6 mg/24 h	20	No
4	45	3 × 30 g	No	No	3	No
5	48	No	No	7,2 mg/24 h	12	No
6	46	5 × 20 g	No	2,4 mg/24 h	13	No
7	58	No	No	No	30	Septic shock
8	55	No	5 cycles of PE	12 mg/24 h	5	No
9	81	5 × 30 g	5 cycles of IA	No	26	No
10	68	No	No	No	1	Septic shock
11	60	No	5 cycles of IA	No	55	No
12	45	No	7 cycles of PE	No	10	No
13	47	No	5 cycles of PE	No	2	No
14	66	No	unknown cycles of PE	No	3	No
15	52	No	6 cycles of PE	No	8	No
16	42	No	3 cycles of PE	No	4	No
17	39	No	5 cycles of PE	No	16	No

*IVIG* intravenous immunoglobulin, *PE* plasma exchange, *IA* immunoadsorption

## Discussion

Here, we investigated clinical features of seronegative MC compared to AChR-MC requiring mechanical ventilation based on our multicenter cohort of MC [2]. In contrast to MuSK-MCs, which are as old as AChR-MCs [5], we found that in seronegative MC, patients were younger but that the time between diagnosis of MG and onset of MC was longer compared to AChR-MC. Interestingly, 8 of 17 seronegative MCs had thymic abnormalities. Although seronegative MCs were less frequently treated with IVIg, there was no difference in other MC treatments. Furthermore, we did not find any difference in baseline characteristics, in the rate of complications or outcome between the patient groups, which is in contrast to more severely affected MuSK MC patients [5].

Seronegative patients represent 10–15% of all MGs. However, there are only very limited data on the clinical management and outcome of MC. In our cohort of MCs, 17 of 250 (6.8%) events were from seronegative patients [2], which may indicate that MC is less prevalent in seronegative patients. Yet, our study was not designed to unambiguously address this question. While MC occurs in most MG patients within the first 2 years after diagnosis [3], seronegative patients in our cohort developed MC significantly later compared to AChR-MG thus suggesting a less severe disease onset in these patients.

Interestingly, LRP4-positive MGs treated at our centers until now never experienced a MC and all retested seronegative patients in our cohort (40%) were negative for LRP4. Moreover, we did not find any publication about a LRP4-MC suggesting that this subgroup is even less severely affected than seronegative MG.

Seronegative MG patients are a heterogeneous group of patients and although we had stringent inclusion criteria, we cannot rule out that some seronegative patients have low-affinity antibodies or would be positive for complement deposition at the neuromuscular junction [10, 11]. Especially the high portion of thymus hyperplasia in our cohort might argue for low-affinity antibodies since thymic pathologies in MG are known to produce AChR-antibodies [12], but 75% of double negative patients (AChR and MuSK) showed lymph node-type infiltrates in thymus similar to AChR-MG [13].

Limitations of this study arise from its retrospective nature and the relatively small sample size. Nevertheless, our study is by far the largest analysis of seronegative MCs and therefore provides important evidence on the treatment of this understudied patient population. Nevertheless, large prospective multicenter studies are needed to further elucidate the character of seronegative myasthenic crisis and whether specific treatment is warranted compared to AChR-MC or MuSK-MC. Another limitation is that antibody tests were done in different labs and therefore false negative or false positive results due to unspecificity of the test technique or positive/negative results near the threshold range cannot be ruled out in every case, like in previous studies in myasthenia gravis.

We conclude that patients with seronegative MC are younger, with a longer course of disease until first crisis needing MV than AChR-MC but that there is no difference in outcome between these patient groups.

## Data Availability

Anonymized data will be made available upon reasonable request.
